# *Interleukin-17A* Gene Variability in Patients with Type 1 Diabetes Mellitus and Chronic Periodontitis: Its Correlation with IL-17 Levels and the Occurrence of Periodontopathic Bacteria

**DOI:** 10.1155/2016/2979846

**Published:** 2016-01-27

**Authors:** Petra Borilova Linhartova, Jakub Kastovsky, Svetlana Lucanova, Jirina Bartova, Hana Poskerova, Jan Vokurka, Antonin Fassmann, Katerina Kankova, Lydie Izakovicova Holla

**Affiliations:** ^1^Clinic of Stomatology, Institutions Shared with St. Anne's Faculty Hospital, Faculty of Medicine, Masaryk University, 656 91 Brno, Czech Republic; ^2^Department of Pathophysiology, Faculty of Medicine, Masaryk University, 625 00 Brno, Czech Republic; ^3^Institute of Clinical and Experimental Dental Medicine, General University Hospital and First Faculty of Medicine, Charles University, 128 08 Prague, Czech Republic

## Abstract

Interleukin-17 contributes to the pathogenesis of type 1 diabetes mellitus (T1DM) and chronic periodontitis (CP). We analyzed* IL-17A* −197A/G and* IL-17F *+7488C/T polymorphisms in T1DM and CP and determined their associations with IL-17 production and occurrence of periopathogens. Totally 154 controls, 125 T1DM, and 244 CP patients were genotyped using 5′ nuclease TaqMan^®^ assays. Bacterial colonization was investigated by a DNA-microarray kit. Production of IL-17 after* in vitro* stimulation of mononuclear cells by mitogens and bacteria was examined by the Luminex system. Although no differences in the allele/genotype frequencies between patients with CP and T1DM + CP were found, the* IL-17A* −197 A allele increased the risk of T1DM (*P* < 0.05). Levels of HbA1c were significantly elevated in carriers of the A allele in T1DM patients (*P* < 0.05). Production of IL-17 by mononuclear cells of CP patients (unstimulated/stimulated by* Porphyromonas gingivalis*) was associated with* IL-17A* A allele (*P* < 0.05).* IL-17A* polymorphism increased the number of* Tannerella forsythia *and* Treponema denticola* in patients with CP and T1DM + CP, respectively (*P* < 0.05).* IL-17A* gene variability may influence control of T1DM and the “red complex” bacteria occurrence in patients with CP and T1DM + CP. Our findings demonstrated the functional relevance of the* IL-17A* polymorphism with higher IL-17 secretion in individuals with A allele.

## 1. Introduction

Type 1 diabetes mellitus (T1DM) is an autoimmune disease caused by T cell-mediated destruction of pancreatic *β* cells resulting in the absence of insulin and uncontrolled hyperglycemia. A complex interplay between genetic and environmental factors participates in developing of T1DM and manifestation of its systemic and oral complications. Periodontitis has been identified as the sixth complication of diabetes; the other five complications are retinopathy (DR), nephropathy (DN), neuropathy (DPN), macrovascular disease, and poor wound healing [1]. Periodontitis is a chronic infection characterized by progressive inflammatory response to bacteria in dental plaque, which finally results in periodontal tissue destruction and tooth loss. The relationship between diabetes mellitus and periodontitis has been reported previously [2]. Chronic hyperglycemia induces a proinflammatory state in the gingival microcirculation characterized by an increased vascular permeability, and leukocyte and endothelial cell activation, which may contribute to periodontal tissue damage in diabetes mellitus [3]. Chronic periodontitis (CP) is more frequent in T1DM patients and can worsen its metabolic control [4, 5]. Nevertheless, molecular mechanisms responsible for periodontal disease and its progression in T1DM patients remain unknown.

Although individuals at risk for T1DM are recognized by screening for HLA-associated risk genotypes and *β* cell autoantibodies, recently the pathogenic role of IL-17-secreting T helper 17 (Th17) cells has been implicated in the development of T1DM [6–8]. The IL-17 cytokine family consists of six cytokine members designed from IL-17A (originally cloned and named CTLA-8), IL-17B, IL-17C, IL-17D, IL-17E, and IL-17F, according to the order of their discoveries. IL-17A and IL-17F, the most studied members in the IL-17 family, are located close to each other on human chromosome 6, sharing the highest amino acid sequence identity (50%) and similar functions. They are primarily involved in autoimmune responses, tumor development, and host defense against bacterial and fungal infections by activating epithelial innate immune responses and production of antimicrobial peptides, cytokines (e.g., IL-6 or TNF-*α*), chemokines (e.g., IL-8), and cartilage degrading metalloproteinases as well as cytokines promoting osteoclastogenesis that results in bone destruction [9–11].

Several lines of evidence suggest that IL-17 plays a role in human diabetes. Bradshaw et al. [12] observed that monocytes isolated from T1DM patients induced more IL-17 producing T cells compared with healthy controls. They also observed significantly increased IL-17 producing T cells in peripheral blood of patients with long standing T1DM [12]. Arif et al. [13] demonstrated that activation of IL-17 pathway accelerated pancreatic *β* cell apoptosis and led to autoimmune diabetes. They observed significantly elevated IL-17A expression in pancreas from newly diagnosed T1DM patients. In addition, peripheral blood lymphocytes from T1DM patients had elevated IL-17A and IL-17F expression [14].

The emerging role of IL-17 in periodontal disease was also discussed in a recent study, based on evidence from human and animal models [15]. Upregulated* IL-17A* gene expression has been observed in patients with CP, suggesting that the net effect of IL-17 signaling promotes the disease development [16–22]. Additionally, IL-17RA deficient mice were found more susceptible to* Porphyromonas gingivalis* (*P.g.*), causal Gram-negative bacteria of the “red complex” [23, 24]. Based on the fact that increased IL-17 levels occur in the gingival tissue of patients with periodontal disease [25–27], Park et al. [28] hypothesized that* P.g.* lipopolysaccharide (LPS) might mediate IL-17 release from human periodontal ligament cells.

Despite the important role of IL-17 cytokine in T1DM and CP pathogenesis, no study has investigated* IL-17 *gene variability in T1DM patients so far and only a few studies have reported a relationship between polymorphisms in the* IL-17* gene and periodontitis with contradictory results [29–33]. Therefore, in the present study, we aimed to investigate the association of* IL-17A* −197A/G (rs2275913) and* IL-17F* +7488C/T (His161Arg, rs763780) gene polymorphisms with T1DM and/or CP. In addition,* IL-17A* single nucleotide polymorphism (SNP) was examined in relation to the occurrence of selected periodontal bacteria in subgingival pockets and production of IL-17 by mononuclear cells in a subgroup of CP patients and healthy controls.

## 2. Material and Methods

The study was performed with the approval of the Committees for Ethics of the Medical Faculty, Masaryk University Brno and St. Anne's Faculty Hospital. Written informed consent was obtained from all participants in line with the Helsinki declaration before inclusion in the study.

### 2.1. Study Population and Clinical Examinations

In this case-control study, 523 unrelated adult subjects from the Czech Republic were included. One hundred and twenty-five patients with T1DM were followed in the outpatient unit of the Diabetology Clinics in South Moravia Region, Czech Republic. The diagnosis of T1DM was originally based on the presence of clinical symptoms (such as polyuria, polydipsia, and weight loss) and biochemical parameters (glycemia, ketoacidosis, and autoantibody status). All patients were receiving intensified insulin therapy or insulin pump and other medicaments according to the presence of diabetic complications, such as DN, DR, DPN, and other comorbidities as described on a part of our cohort previously [34]. Duration of diabetes was defined as the period from diabetes onset until the enrolment in this study. Levels of glycemia, glycated hemoglobin (HbA1c), total cholesterol, triglycerides, high density lipoprotein (HDL), low density lipoprotein (LDL), body mass index (BMI), and further parameters were recorded. The periodontal status was evaluated in a subgroup of 38 diabetic patients, 154 healthy controls, and 244 CP subjects recruited from a patient pool of the Clinic of Stomatology, St. Anne's Faculty Hospital Brno, from 2005 to 2015. The controls were selected from subjects referred to the Clinic of Stomatology for reasons other than periodontal disease (such as dental caries, orthodontic consultations, and preventive dental checkups) during the same period as patients and matched for age and gender. Exclusion criteria were history of systemic diseases such as cardiovascular disorders (e.g., coronary artery diseases), diabetes mellitus, malignant diseases, immunodeficiency, and current pregnancy or lactation.

The diagnosis of periodontitis/nonperiodontitis was based on the detailed clinical examination, medical and dental history, tooth mobility, and radiographic assessment as described in our previous study [35].

### 2.2. Genetic Analysis

Isolation and storage of DNA and genotyping of samples were conducted in the laboratory of the Department of Pathophysiology, Faculty of Medicine, Masaryk University, Brno, Czech Republic.

Genotyping of two SNPs in* IL-17*,* IL-17A* −197A/G (rs2275913), and* IL-17F *+7488C/T (His161Arg, rs763780) was based on polymerase chain reaction using 5′ nuclease TaqMan assays (C_15879983_10, C_2234166_10, resp.). Reaction mixture and conditions were designed according to the manufacturer's instructions (Thermo Fisher Scientific, Waltham, MA, USA) and fluorescence was measured using the ABI PRISM 7000 Sequence Detection System. SDS version 1.2.3 software was used to analyze real-time and endpoint fluorescence data.

### 2.3. DNA-Microarray Analyses of Oral Pathogens

DNA-microarray analyses of oral pathogens based on a periodontal pathogen detection kit (Protean Ltd., Ceske Budejovice, Czech Republic) were previously used and described [35]. Bacterial colonization [*Aggregatibacter actinomycetemcomitans *(*A.a.*),* Tannerella forsythia *(*T.f.*),* P.g.*,* Prevotella intermedia *(*P.i.*),* Treponema denticola *(*T.d.*),* Peptostreptococcus micros *(*P.i.*), and* Fusobacterium nucleatum *(*F.n.*)] in subgingival sulci/pockets was investigated in a subgroup of controls (*N* = 51), CP patients (*N* = 182), and T1DM patients with CP (*N* = 38) before subgingival scaling. This test determined the individual pathogens semiquantitatively as follows: (−) undetected, which corresponds to the number of bacteria less than 10^3^, (+) slightly positive corresponding to the number of bacteria 10^3^ to 10^4^, (++) positive corresponding to the number of bacteria 10^4^ to 10^5^, and (+++) strongly positive, with the number of bacteria higher than 10^5^.

### 2.4. Cultivation of Bacteria and Immunological Examination

Cultivation of periodontopathic bacteria and immunological examination were performed in the laboratory of the Institute of Clinical and Experimental Dental Medicine, General University Hospital and First Faculty of Medicine, Charles University, Prague, Czech Republic.

IL-17 levels were measured in a subgroup of 15 healthy controls and 30 patients with CP. IL-17 levels were determined in mononuclear cells isolated from 20 mL of heparinized blood using the Luminex multiplex method (Luminex 100TM analyzer, R&D systems, USA). The isolation, cultivation, and stimulation of cells by selected bacteria (*A.a.*,* T.f.*,* P.g.*, and* P.i.*) and mitogens or Heat Shock Protein (HSP) 70 were described in detail in our previous article [36].

### 2.5. Statistical Analysis

Statistical analysis was performed using the statistical package Statistica v. 10 (StatSoft Inc., USA). Standard descriptive statistics were applied in the analysis: absolute and relative frequencies for categorical variables and mean with standard deviation (SD) or median with quartiles for quantitative variables. To compare independent groups, one-way analysis of variance (ANOVA) and Kruskal-Wallis (ANOVA) were performed to compare continuous variables. The allele frequencies were calculated from the observed numbers of genotypes. The differences in the allele frequencies were tested by the Fisher-exact test; Hardy-Weinberg equilibrium (HWE) and genotype frequencies were calculated by the chi-square test (*χ* ^2^). The association was described by odds ratios (OR) with 95% confidence intervals (95% CI). Only the values of *P* less than 0.05 were considered as statistically significant.

## 3. Results

### 3.1. Case-Control Study

The demographic data of the study population are shown in Table 1. The mean ages and BMI between the healthy controls and patient groups did not differ significantly (*P* > 0.05). There were also no significant differences between the subjects with T1DM or CP and the controls relating to males/females ratio or smoking status. All diabetic patients (*N* = 38) who were examined at the Periodontology Department were affected by periodontitis. Duration of DM and diabetic complications (DN, DR, and DPN) in the whole diabetic cohort versus subgroup of T1DM patients with CP were not statistically different.

### 3.2. SNPs Analysis

Both studied polymorphisms were in the HWE in the control group (*P* > 0.05). Considering the fact that, in the Czech population, SNP* IL-17F* +7488C/T (His161Arg, rs763780) TT genotype occurred in 93.7%, we analyzed this polymorphism only in the subgroup of subjects (*N* = 190, data not shown).

The allele and genotype frequencies of the* IL-17A* −197A/G (rs2275913) variant are shown in Table 2. While the genotype frequencies were not different between the controls and CP or T1DM patients, the A allele was marginally associated with an increased risk to T1DM (*P* < 0.05, OR = 1.36, 95% CI = 0.96–1.92). Moreover, mean levels of HbA1c were significantly elevated in carriers of the A allele (AA and AG genotypes in comparison to GG homozygotes) in a group of T1DM patients (76.6 mmol/mol versus 69.8 mmol/mol, *P* < 0.05, Table 3).

Although no significant differences in the* IL-17A* allele or genotype frequencies between patients with CP and healthy controls were found in the whole set (Table 2), stratification of subjects according to smoking status revealed the following differences: in healthy subjects, the A allele frequency was higher in smokers (*P* < 0.05, OR = 1.62, 95% CI = 0.97–2.70), whereas, in CP patients, this allele was found more frequently in nonsmokers (*P* < 0.05, OR = 0.64, 95% CI = 0.41–1.02). Although a similar distribution of alleles or genotypes between the groups of control nonsmokers versus CP nonsmokers was found, frequencies of the A allele (*P* < 0.01, OR = 2.14, 95% CI = 1.19–3.83), AA genotype (*P* < 0.05, OR = 3.17, 65% CI = 0.89–11.28), and AA + AG versus GG genotypes (*P* < 0.05, OR = 1.82, 95% CI = 0.81–4.09) were more frequent in the control smokers in comparison with the smokers with CP (Table 4). However, as the associations detected between the given subgroups were only of borderline significance and numbers of the individuals in the subgroups were small, the results obtained should be interpreted carefully. A subanalysis performed separately in the groups of females (*N* = 269) and males (*N* = 254) showed no significant difference in the* IL-17A* −197A/G (rs2275913) allele or genotype frequencies (data not shown). Due to the small number of T1DM patients with confirmed CP, this group was not analyzed according to gender or smoking status.

### 3.3. Microbial Analysis

Possible links between the* IL-17A* −197A/G (rs2275913) variant and the occurrence of seven selected periodontal bacteria in subgingival pockets were analyzed. The* IL-17A* −197 A allele carriers had an increased risk of* T.f. *occurrence in 182 CP patients (34.9% versus 19.6%, *P* < 0.05, OR = 2.20, 95% CI = 1.03–4.73) and a similar but nonsignificant trend was observed for the presence of* P.i.* (36.0% versus 28.7%, *P* = 0.09, OR = 1.40, 95% CI = 0.89–2.20). On the other hand, in patients with T1DM and CP carrying A allele of the* IL-17A* −197 polymorphism,* T.d.* in subgingival biofilm occurred less frequently than in subjects without this allele (26.9% versus 50.0%, *P* < 0.05, OR = 0.37, 95% CI = 0.13–1.01, Table 5). In subgroups of nonsmokers, the* IL-17A* −197 A allele carriers had an increased risk for the occurrence of* P.i.* in patients with CP only (39.6% versus 28.7%, *P* < 0.05, OR = 1.63, 95% CI = 0.96–2.76, Table 5). The* IL-17A* −197 SNP was not associated with the occurrence of any other bacteria (including* P.g.*), from those seven selected ones (data not shown).

### 3.4. Immunological Analysis

We analyzed IL-17 levels in relation to the* IL-17A* −197A/G (rs2275913) polymorphism in selected patients with CP and healthy controls. The IL-17 levels were measured after a 3-day* in vitro* cultivation of mononuclear cells, without or with stimulation by dental plaque bacteria, mitogens, or HSP70 in the CP patients (*N* = 30). Carriers of genotype with the A allele of* IL-17A* −197A/G (rs2275913) SNP had a higher production of IL-17 by unstimulated monocytes (0.98 pg/mL versus 0.27 pg/mL, *P* < 0.05) and also after stimulation with* P.g.* (1.51 pg/mL versus 0.10 pg/mL, *P* < 0.05) than GG homozygotes (Table 6). In the healthy controls (*N* = 15), unstimulated IL-17 levels were 0.10 (0.08–0.26) pg/mL (median; 25–75 quartiles) without any significant relationship with* IL-17A* polymorphism. After pooling both groups (*N* = 45), production of IL-17 was significantly associated with* IL-17A* polymorphism in the unstimulated mononuclear cells (*P* < 0.05), but not after stimulation by* P.g.* (*P* = 0.06, data not shown).* IL-17A* −197A/G SNP had no significant effect on IL-17 production after stimulation with other periodontal bacteria, HSP70, and/or mitogens (data not shown).

## 4. Discussion

Inflammation is a physiological immune response triggered during infection and injury in an attempt to prevent infection and promote regeneration. However, persistent and unwarranted inflammation can result in host tissue damage [8]. Regulation of inflammation is a complex process, tightly controlled by signaling messengers of the immune system, such as cytokines. IL-17A and IL-17F, produced mainly by Th17 cells, have been found to be involved in the pathogenesis of autoimmune diseases including diabetes and chronic inflammation, such as periodontitis [12, 15, 37–41].

We evaluated* IL-17A* −197A/G (rs2275913) and* IL-17F* +7488C/T (His161Arg, rs763780) SNPs in a group of adults with and without T1DM and/or CP from Czech population. As the present study identified a low variability of* IL-17F* at position +7488C/T (similarly as other studies in other populations: http://www.snpedia.com/index.php/Rs763780), the TT genotype occurred in 93.7%, and no CC homozygote was detected, we investigated this polymorphism only in a subgroup of 190 subjects. Further, no associations of this polymorphism with aggressive periodontitis (AgP) or CP [31–33] have been previously found. In contrast, the* IL-17A* −197A/G SNP was analyzed in the whole set of our 523 subjects. The minor allele frequency (MAF) of this SNP was 0.36, which is in line with the allele frequency in other European population [42]. To this date, no study investigating an association of the* IL-17A* −197A/G or* IL-17F* +7488C/T variants with T1DM or periodontitis in European white population has been published. Although no significant differences were found in the genotype frequencies between the healthy subjects and T1DM or CP patients, the A allele was marginally associated with an increased risk of T1DM (*P* < 0.05). This allele displayed a higher affinity for the nuclear factor of activated T cells (NFAT), a critical transcription factor involved in the IL-17 regulation [43, 44]. Espinoza et al. [43] reported that healthy individuals possessing the A allele of* IL-17A* −197A/G (rs2275913) produced significantly more IL-17 after* in vitro* T cells stimulation than those without this allele. Shao et al. [45] described that uncontrolled expansion of Th17 cells was involved in T1DM pathology and might exert essential effects on its development.

In the studied diabetic population, significantly higher levels of HbA1c were present in T1DM carriers of the genotype with the A allele (AA + AG genotypes) versus the GG homozygotes (76.6 mmol/mol ± 16.5 mmol/mol versus 69.8 mmol/mol ± 13.9 mmol/mol). To our knowledge, no previous study has focused on this issue; however, polymorphisms in other genes, for example,* IL-6* [46] and* IL-18* separately [47] or in combination with the* IL-12B* gene [48], have been associated with higher concentration of HbA1c in T1DM populations.

In addition, we found no significant differences in the* IL-17A* −197A/G (rs2275913) allele or genotype frequencies between the healthy subjects and patients with CP, not even after stratification by sex (data not shown). No studies on this topic from European but only from Iranian and Brazilian populations have been reported. In the Iranian population, the CC genotype of another* IL-17A* variant (rs10484879) was associated with CP and peri-implantitis [29]. Three Brazilian studies examined variability in the* IL-17A* −197A/G (rs2275913) gene in relation to periodontal disease with controversial results. In the study by Saraiva et al. [32], the A allele was associated with the absence of periodontitis, but Corrêa et al. [31] and Zacarias et al. [33] found the AA genotype and the A allele as a risk factor for CP. Even in the separate subgroup of Czech patients with CP and T1DM, no relationship between* IL-17A* polymorphism and periodontal status (PD = probing pocket depth, CAL = clinical attachment loss, etc.) was found. However, Gürsoy et al. [49] recently described the association between PD and IL-17 levels in saliva of type 2 diabetic patients, but independently of glycemic status. Interestingly, the allele and genotype distributions of the* IL-17A* variant in the subgroup of T1DM patients with CP were closer to the values in nondiabetic CP than T1DM patients. The different findings in Czech population may be due to differences in European versus Brazilian populations.

In contrast to findings in the Brazilian cohort where the AA genotype was identified as a risk factor for CP in nonsmoker Caucasians [33], our results showed no differences in distribution of the* IL-17A* −197A/G alleles or genotypes between healthy and periodontitis nonsmokers. However, the G allele and the GG genotype were marginally significantly associated with an increased risk of periodontitis in smokers. Analysis of the allele frequencies in nonsmokers versus smokers showed borderline significant differences in both studied groups but in the opposite trend, in which 68.1% versus 56.8% for healthy controls and 64.5% versus 73.8% for patients with CP. Due to a relatively small number of subjects in the individual subgroups, number of comparisons performed, and only marginally significant differences, our results should be interpreted carefully.

In the next step, according to the hypothesis about biological functions and regulation of IL-17 which plays a role in host defense [50], we assessed the* IL-17A* gene variability in relation to the presence of periodontopathic bacteria in subgingival pockets in patients with CP and T1DM + CP. In CP population,* IL-17A* −197 A allele carriers had an increased risk of* T.f. *occurrence and, in CP nonsmokers only, this allele increased risk of the occurrence of* P.i.*, but the same A allele was protective for the presence of* T.d.* in subgingival biofilm in T1DM patients with CP. In connection with periodontopathic bacteria, gene variability has been investigated in a few other interleukins to this date. The specific* IL-8* variants were associated with subgingival colonization with* A.a. *in AgP and* T.f. *in CP in the Czech population [35]. Finoti et al. [51] also found that* IL-8* haplotype influenced the presence of the “red complex” bacteria in gingival sulci. Several studies by Nibali et al. [52–56] focused on a correlation between the pathogenic bacterial colonization and variability in* IL-6* and* Fc γ receptor* genes. Additionally, SNPs in the* IL-1 *gene cluster [57],* interferon γ*, and* IL-2* were associated with the presence of various periodontopathic bacteria, especially* A.a.* and the “red complex” bacteria. However, in our study, no other bacteria from those studied were associated with* IL-17A* −197 variant and there were no significant differences in the occurrence of these bacteria among groups.

Finally, in functional study, we associated IL-17 production* in vitro *by blood mononuclear cells with the* IL-17A* −197A/G (rs2275913) gene polymorphism in the subgroup of patients with CP. In Czech periodontitis patients,* IL-17A* −197 AA + AG carriers had higher IL-17 levels in unstimulated mononuclear cells than GG homozygotes. Our results are in accordance with the conclusion of Espinoza et al. [43], who connected the A allele with increased IL-17 levels* in vitro* after T cells stimulation. Even greater differences in IL-17 production were measured in CP carriers with the A allele in genotype after stimulation with* P.g*. It is in agreement with previous findings that* P.g.* LPS is a mediator of IL-17 release from human periodontal ligament cells [28]. Also Moutsopoulos et al. [58] proved that* P.g.* induced innate cell IL-17 production and promoted Th17 polarization. In a pooled group of CP and healthy subjects (*N* = 45), production of IL-17 was associated with the* IL-17A* polymorphism only in unstimulated mononuclear cells, but not after stimulation by* P.g.*, other periodontal bacteria, HSP70, and/or mitogens. In addition, recently Azman et al. [20] demonstrated that serum, saliva, and gingival crevicular fluid, IL-17A levels were higher in periodontitis patients and correlated positively with clinical parameters (PD, CAL, and BOP = bleeding on probing). Findings of the present study also demonstrated increased IL-17 levels in unstimulated monocytes in patients with periodontitis versus healthy controls (0.48 pg/mL versus 0.10 pg/mL; median). However, the comparison of absolute values of IL-17 levels in healthy subjects with results of other studies would require the use of the same method for the cytokine determination (including a kit from the same supplier), type and preparation of samples, and age of subjects [59, 60].

There are many possible limitations in this study. The present study is mainly limited by relatively low numbers of subjects and especially by the fact that from the group of 125 T1DM subjects, only 38 patients were evaluated for periodontal status. The negative findings of single marker analysis in this subgroup could be a result of a lack of statistical power to detect minor differences (small effect of genes/gene variants in multifactorial diseases is typical). In addition, most of the associations found were tightly below statistical significance (*P* < 0.05) without multitest corrections. Therefore, risk of relatively high false discovery rate means that our results should be interpreted with caution. Secondly, levels of IL-17 in plasma or in gingival tissue were not measured and the presence of periodontal pathogens was examined only in the 271 subjects. However, this study also has several strengths. This is the first study of the* IL-17 *gene polymorphisms in T1DM population and in European patients with CP that was conducted in a relatively homogenous population of white Caucasians in Central Europe of the Czech origin. Secondly, the size of studied healthy subjects and CP patients is greater than in previous Iranian or Brazilian studies. Thirdly, we examined not only the polymorphisms alone but their relationship with clinical, bacterial, and biochemical parameters, which allowed a better biological assessment of the detected associations.

## 5. Conclusions

In conclusion,* IL-17A* gene variability may partially influence T1DM control and the “red complex” bacteria occurrence in patients with CP and diabetic patients with CP. Additionally, our findings confirmed the functional relevance of the* IL-17A* polymorphism with higher IL-17 secretion in the individuals with the A allele. However, the results of this study need to be proven in a larger independent cohort.

## Figures and Tables

**Table 1:** Demographic data of the healthy controls and the studied subjects with CP, T1DM (and the T1DM + CP subgroup).

Characteristics	Controls *N* = 154	CP *N* = 244	T1DM *N* = 125	T1DM + CP *N* = 38
Age (mean years ± SD)	48.5 ± 10.7	52.5 ± 9.8	46.4 ± 13.8	49.9 ± 10.6
Sex (males/females)	75/79	112/132	67/58	16/22
Smoking (no/yes, %)	71.1/28.9	73.9/26.1	78.9/21.1^#^	78.9/21.1
BMI (mean ± SD)	23.2 ± 4.6	26.4 ± 3.7	25.0 ± 4.9	25.3 ± 3.1
Duration of DM (mean years ± SD)	—	—	22.8 ± 10.3	24.0 ± 11.3
HbA1c (mmol/mol, mean ± SD)	—	—	76.5 ± 17.3	69.9 ± 11.4
DN (no/yes, %)	—	—	52.9/47.1	71.9/28.1
DR (no/yes, %)	—	—	30.4/69.6	42.4/57.6
DPN (no/yes, %)	—	—	39.3/60.7	47.1/52.9

BMI = body mass index, CP = chronic periodontitis, DN = diabetic nephropathy, DPN = diabetic peripheral neuropathy, DR = diabetic retinopathy, HbA1c = glycated hemoglobin, *N* = number of subjects, SD = standard deviation, and T1DM = type 1 diabetes mellitus.

^#^Smoking status is known only in T1DM patients with CP.

**Table 2:** *IL-17A *−197A/G (rs2275913) genotype and allele frequencies in healthy controls, patients with CP, T1DM (and the T1DM + CP subgroup).

*IL-17A* −197	Controls	CP			T1DM			T1DM + CP		

Genotype and allele	*N* = 154 (%)	*N* = 244 (%)	*P*	OR (95% CI)	*N* = 125 (%)	*P*	OR (95% CI)	*N* = 38 (%)	*P*	OR (95% CI)

AA	18 (11.7)	32 (13.1)	0.56	1.00 (0.52–1.93)	23 (18.4)	0.05	1.93 (0.93–4.00)	6 (15.8)	0.46	1.20 (0.42–3.48)
AG	71 (46.1)	97 (39.8)	0.14	0.77 (0.50–1.19)	59 (47.2)	0.23	1.26 (0.75–2.11)	14 (36.8)	0.25	0.71 (0.33–1.55)
GG	65 (42.2)	115 (47.1)		1.00	43 (34.4)		1.00	18 (47.4)		1.00

AA + AG versus GG	89 versus 65 (57.8/42.2)	129 versus 115 (52.9/47.1)	0.20	0.82 (0.55–1.23)	82 versus 43 (65.6/34.4)	0.11	1.39 (0.85–2.27)	20 versus 18 (52.6/47.4)	0.35	0.81 (0.40–1.65)

A	107 (34.7)	161 (33.0)		0.92 (0.68–1.25)	105 (42.0)		1.36 (0.96–1.92)	26 (34.2)		0.98 (0.58–1.66)
G	201 (65.3)	327 (67.0)	0.33	1.00	145 (58.0)	0.05^*∗*^	1.00	50 (65.8)	0.37	1.00

CI = confidential interval, CP = chronic periodontitis, *N* = number of subjects, OR = odds ratio, and T1DM = diabetes mellitus type 1; ^*∗*^ *P* value < 0.05 (in comparison with healthy controls).

**Table 3:** Levels of HbA1c in correlation with *IL-17A *−197A/G (rs2275913) genotypes in T1DM^#^.

*IL-17A* −197	T1DM		

Genotype	*N*	HbA1c (mmol/mol)	*P*
		Mean ± SD	

AA + AG	69	76.6 ± 16.5	
GG	35	69.8 ± 13.9	0.03^*∗*^

HbA1c = glycated hemoglobin, *N* = number of subjects, SD = standard deviation, and T1DM = type 1 diabetes mellitus; ^*∗*^ *P* value < 0.05 (parametric test, ANOVA).

^#^Levels of HbA1c were available in 104 patients with T1DM.

**Table 4:** *IL-17A *−197A/G (rs2275913) genotype and allele frequencies in healthy controls and patients with CP stratified by smoking status.

*IL-17A* −197	Control nonsmokers	Control smokers		CP nonsmokers	CP smokers			

Genotype and allele	*N* = 108 (%)	*N* = 44 (%)	*P*	*N* = 173 (%)	*N* = 61 (%)	*P*	*P* ^*a*^	*P* ^*b*^

AA	10 (9.2)	8 (18.2)		28 (16.2)	4 (6.6)			
AG	49 (45.4)	22 (50.0)		67 (38.7)	24 (39.3)			
GG	49 (45.4)	14 (31.8)	0.16	78 (45.1)	33 (54.1)	0.15	0.22	0.04^*∗*^
AA + AG versus GG	59 versus 49 (54.6/45.4)	30 versus 14 (68.2/31.8)	0.09	95 versus 78 (54.9/45.1)	28 versus 33 (45.9/54.1)	0.14	0.10	0.02^*∗*^
A	69 (31.9)	38 (43.2)		123 (35.5)	32 (26.2)			
G	147 (68.1)	50 (56.8)	0.04^*∗*^	223 (64.5)	90 (73.8)	0.04^*∗*^	0.22	0.01^*∗*^

CP = chronic periodontitis, *N* = number of subjects, and ^*∗*^ *P* value < 0.05.

*P* ^*a*^ comparison of groups of nonsmokers.

*P* ^*b*^ comparison of groups of smokers.

**Table 5:** The presence of bacteria in correlation with *IL-17A *−197A/G (rs2275913) polymorphism in patients with CP and T1DM + CP^#^.

*IL-17A* −197	CP			CP			T1DM + CP		
	*N* = 182 (%)			*N* = 182 (%)			*N* = 38 (%)		

Genotype and allele	*T.f.* neg	*T.f.* pos	*P*	*P.i.* neg	*P.i.* pos	*P*	*T.d.* neg	*T.d.* pos	*P*

AA	0 (0.0)	24 (15.1)	0.02^*∗*^	6 (8.0)	18 (16.8)	0.07	3 (25.0)	3 (11.5)	0.14
AG	9 (39.1)	63 (39.6)	0.33	31 (41.3)	41 (38.3)	0.51	6 (50.0)	8 (30.8)	0.11
GG	14 (60.9)	72 (45.3)		38 (50.7)	48 (44.9)		3 (25.0)	15 (57.7)	

AA + AG versus GG	9 versus 14 (39.1 versus 60.9)	87 versus 72 (54.7 versus 45.3)	0.12	37 versus 38 (49.3 versus 50.7)	59 versus 48 (55.1 versus 44.9)	0.27	9 versus 3 (75.0 versus 25.0)	11 versus 15 (42.3 versus 57.7)	0.06

A	9 (19.6)	111 (34.9)		43 (28.7)	77 (36.0)		12 (50.0)	14 (26.9)	

G	37 (80.4)	207 (65.1)	0.03^*∗*^	107 (71.3)	137 (64.0)	0.09	12 (50.0)	38 (73.1)	0.04^*∗*^

*IL-17A* −197	CP nonsmokers			CP nonsmokers			T1DM + CP nonsmokers		
	*N* = 131 (%)			*N* = 131 (%)			*N* = 30 (%)		

Genotype and allele	*T.f.* neg	*T.f.* pos	*P*	*P.i.* neg	*P.i.* pos	*P*	*T.d.* neg	*T.d.* pos	*P*

AA	0 (0.0)	20 (17.5)	0.06	4 (7.4)	16 (20.8)	0.04^*∗*^	2 (25.0)	3 (13.6)	0.27

AG	8 (47.1)	44 (38.6)	0.59	23 (42.6)	29 (37.7)	0.51	4 (50.0)	7 (31.8)	0.21

GG	9 (52.9)	50 (43.9)		27 (50.0)	32 (41.6)		2 (25.0)	12 (54.5)	

AA + AG versus GG	8 versus 9 (47.1 versus 52.9)	64 versus 50 (56.1 versus 43.9)	0.33	27 versus 27 (50.0 versus 50.0)	45 versus 32 (58.5 versus 41.6)	0.22	6 versus 2 (75.0 versus 25.0)	10 versus 12 (45.4 versus 54.5)	0.15

A	8 (23.5)	84 (36.8)		31 (28.7)	61 (39.6)		8 (50.0)	13 (29.5)	

G	26 (76.5)	144 (63.2)	0.09	77 (71.3)	93 (60.4)	0.05^*∗*^	8 (50.0)	31 (70.5)	0.12

CP = chronic periodontitis, *N* = number of subjects, neg = negative, pos = positive, T1DM = type 1 diabetes mellitus, *P.i.* = *Prevotella intermedia*,* T.d.* = *Treponema denticola*,* T.f.* = *Tannerella forsythia*, and ^*∗*^ *P* value < 0.05.

^#^Of the seven periodontal pathogens analyzed, only those with significant differences are shown.

**Table 6:** Levels of IL-17 in correlation with *IL-17A *−197A/G (rs2275913) genotypes in patients with CP^#^.

*IL-17A* −197	IL-17 unstimulated (pg/mL); median (25–75 quartiles)		

Genotype	*N*		*P*

AA + AG	19	0.98 (0.25–8.53)	
GG	11	0.27 (0.00–0.54)	0.04^*∗*^

*IL-17A* −197	IL-17 stimulated by *P.g.* (pg/mL); median (25–75 quartiles)		

Genotype	*N*		*P*

AA + AG	19	1.51 (0.50–4.56)	
GG	11	0.10 (0.00–1.51)	0.02^*∗*^

CP = chronic periodontitis, *N* = number of subjects, ^*∗*^ *P* value < 0.05 (Kruskal-Wallis test, ANOVA), and *P.g.* = *Porphyromonas gingivalis.*

Levels of IL-17 were available in 30 patients with CP.

^#^Only significant differences were shown.
